# Identification of variation in nutritional practice in neonatal units in England and association with clinical outcomes using agnostic machine learning

**DOI:** 10.1038/s41598-021-85878-z

**Published:** 2021-03-30

**Authors:** Sam F. Greenbury, Kayleigh Ougham, Jinyi Wu, Cheryl Battersby, Chris Gale, Neena Modi, Elsa D. Angelini

**Affiliations:** 1 NIHR Imperial Biomedical Research Centre, ITMAT Data Science Group, Imperial College London, London, UK; 2 Department of Metabolism, Digestion and Reproduction, Imperial College London, London, UK; 3 Section of Neonatal Medicine, School of Public Health, Imperial College London, Chelsea and Westminster Hospital Campus, London, UK

**Keywords:** Health care, Biomedical engineering, Computational science

## Abstract

We used agnostic, unsupervised machine learning to cluster a large clinical database of information on infants admitted to neonatal units in England. Our aim was to obtain insights into nutritional practice, an area of central importance in newborn care, utilising the UK National Neonatal Research Database (NNRD). We performed clustering on time-series data of daily nutritional intakes for very preterm infants born at a gestational age less than 32 weeks (n = 45,679) over a six-year period. This revealed 46 nutritional clusters heterogeneous in size, showing common interpretable clinical practices alongside rarer approaches. Nutritional clusters with similar admission profiles revealed associations between nutritional practice, geographical location and outcomes. We show how nutritional subgroups may be regarded as distinct interventions and tested for associations with measurable outcomes. We illustrate the potential for identifying relationships between nutritional practice and outcomes with two examples, discharge weight and bronchopulmonary dysplasia (BPD). We identify the well-known effect of formula milk on greater discharge weight as well as support for the plausible, but insufficiently evidenced view that human milk is protective against BPD. Our framework highlights the potential of agnostic machine learning approaches to deliver clinical practice insights and generate hypotheses using routine data.

## Introduction

Neonatal care is a common, high-cost clinical service. In the UK and other high-income countries, around one in seven newborn infants will be admitted to a neonatal unit^1,2^. Within the National Health Service (NHS) in England it is among the top three high-cost nationally commissioned services. Neonatal care encompasses a broad range of conditions and clinical processes ranging from full intensive care for the most preterm and critically unwell infants to minimal additional support. Length of stay for very preterm infants (gestational age < 32 weeks) is high, and commonly over 100 days in infants born at < 28 weeks gestation^3^. Clinical care for very preterm infants comprises multiple treatment components that vary on a daily or more frequent basis. Preterm birth is responsible for around 40% of under-five deaths globally and can have life-long impact^4,5^. Describing the complex, time varying, neonatal care practices is an essential first step in understanding and potentially influencing short- and long-term outcomes.

Nutritional practice for preterm infants^6^ is challenging due to immature organ systems and multiple co-morbidities, and varies greatly^7^ despite clinical guidelines, in large part because of an inadequate evidence-base^8^. Many factors affect nutritional care^9^, and decisions involve clinicians, parents, nurses and other healthcare professionals. Maternal milk is recommended as the optimal source of nutrition for very preterm infants^8^. There is strong effort to ensure mothers delivering preterm are supported to express breast milk. In England 95% of very preterm infants receive some maternal milk in the first 14 postnatal days^10^ and around 60% are receiving maternal milk at discharge^11^. Nutritional practice is believed to influence important neonatal outcomes, though strong evidence of causal relationships is lacking. Observational studies have examined the association between nutritional practices and necrotising enterocolitis (NEC), an acquired inflammatory gastrointestinal disease^10^ and bronchopulmonary dysplasia (BPD), a chronic respiratory condition^12^, both major causes of preterm mortality and morbidity. To our best knowledge, machine learning has not previously been used to investigate neonatal nutritional practices.

The availability of large scale real-world data and the advent of machine learning techniques provide the opportunity to augment traditional approaches for generating evidence and improving clinical practice^13,14^. Recent studies indicate that the application of such approaches to clinical data at scale can help identify complex patient characteristics most relevant for making a correct diagnosis^15^, recommending a treatment regimen^15^ or deriving a prognostic model^16^. The use of machine learning to generate models mixing clinical variables and treatment regimens offers the dual advantage of understanding diversity within common clinical profiles or treatment practices, and enabling patient stratification to tailor further models such as risk scoring^17–19^, outcome prediction^20^ or decision support^21,22^. Machine learning models are efficient at clustering^23^ based on the notion of “patient similarity”^24^. In particular, probabilistic models are able to discover clusters and learn interpretable parameters of an underlying generative model that explains the stratification. Traditional unsupervised models, agnostic of any target end-point measure, have seldom been exploited for data-mining of electronic health records (EHR)^25–27^. Unsupervised deep learning^20,28,29^ is rapidly developing to explore EHR, with methods such as autoencoders being utilised to learn “deep patient” representations. While these can facilitate improved accuracy in downstream supervised learning tasks (e.g. predicting mortality), such approaches can suffer from difficulty in interpretability, vital for clinical value, due to the “black-box” nature of the learned representations^30^. In this work, we focus on the application of a probabilistic unsupervised generative model, namely the Dirichlet Process Gaussian Mixture Model (DPGMM)^31,32^, to simultaneously learn, within a large neonatal database of routine clinical data, the number and composition of nutritional clusters, allowing the learned structure of the model to be readily interpretable.

Using the DPGMM approach on data held in the UK National Neonatal Research Database (NNRD), we encoded complex clinical records from very preterm infants to derive clusters that identify clinically comprehensible patterns of nutritional support (*nutritional patterns*) and associated outcomes. We evaluated our results with respect to reproducibility, clinical plausibility, and utility.

Finally, to demonstrate the value of such an approach to inform future randomised evaluations or to investigate causal inference for associations not amenable to interventional studies, we used a recently introduced deconfounding causal inference method to identify associations between patterns of nutritional support and outcomes^33,34^.

## Results

Results are reported over the population of very and extremely preterm infants (gestational age < 32 weeks at birth) born in England between 01/01/2012 and 01/07/2018, comprising 45,679 infants (54% boys; 46% girls) and 2,535,062 days of care, with a median length of stay (LoS) of 49 days. The following daily nutritional variables/components were studied, enteral: Maternal Milk (MM), pasteurised Human Donor Milk (HDM), Breast Milk Fortifier (BMF) and Formula Milk (FM); and parenteral: Parenteral Nutrition (PN) and Glucose Electrolyte solution (GE).

### Descriptive statistics on the cohort of very preterm infants

The median (lower quartile, upper quartile) gestational age (GA) and birth-weight z-score (BW z-score) were 29 (27, 30) weeks and − 0.16 (− 0.78, 0.36) standardised units respectively. Of the cohort, 8% (n = 3,630) received advanced resuscitation (chest compressions or resuscitation drugs) at birth and 90% (n = 40,798) any antenatal steroids.

The median discharge postmenstrual age of the cohort was 36.7 weeks. In terms of outcome characteristics, median (lower quartile, upper quartile), weight z-score at 36 weeks postmenstrual age (W36 z-score) and change in weight z-score from birth to 36 weeks postmenstrual age (W36dz) were − 1.55 (− 2.19, − 0.94) and − 1.16 (− 1.63, − 0.73) respectively. Overall mortality was 8% (n = 3,825); 3% (n = 1,484)developed severe NEC and 27% (n = 12,407) developed BPD (see Methods for the definitions used for NEC and BPD).

The proportion of infants who received each nutritional component on at least a single day shows great variability between GA groups (Table 1), except for GE where for each gestational age figures are over 90%. Across the cohort, considering only enteral nutrition, 15.6% (n = 7,109) of infants were solely fed with MM, 3.3% (n = 1,492) solely with FM, 49.7% (n = 22,684) with a mixture of MM and FM, and 27.4% (12,522) received some HDM. Of the whole cohort, 4.1% (1,872) received no enteral nutrition during their stay. In the first two days of admission, 31.5% (n = 14,367) received MM, while in the final two days prior to discharge 53.7% (n = 24,509) received MM on at least one of those days. Only 1.2% (n = 560) of infants did not receive any PN or GE; of these 82 were fed solely with MM, 57 solely with FM and 421 with a combination. Overall, 45.3% (n = 20,688) of infants received some BMF during their neonatal stay.

**Table 1:** Receipt of each nutritional component stratified by gestational age.

Gestational age (number of infants)	Proportion of infants having received each nutritional component on at least one day during neonatal unit stay						
	MM (%)	HDM (%)	BMF (%)	FM (%)	PN (%)	GE (%)	Survival (%)
22 (55)	47	20	22	22	76	95	27
23 (1,217)	72	27	39	43	88	95	45
24 (2,277)	84	34	53	59	94	97	65
25 (2,607)	89	35	62	68	96	96	79
26 (3,304)	92	36	63	71	96	96	86
27 (4,239)	93	35	61	76	97	95	92
28 (5,599)	94	32	57	76	96	95	93
29 (6,555)	94	30	48	77	92	93	97
30 (8,503)	93	24	39	79	72	93	98
31 (11,323)	91	18	27	82	51	94	98
Total (45,679)	91	27	45	76	80	94	92

Proportions of infants who received each component on at least one day are reported along with survival rates. Components are abbreviated as MM: Maternal milk, HDM: Human Donor Milk, BMF: Breast Milk Fortifier, FM: Formula Milk, PN: Parenteral Nutrition, GE: Glucose Electrolyte solution.

For the lowest gestational age group (GA = 22 weeks), it would be expected that the majority of deaths would occur before BMF is commonly commenced; 80% of GA = 22 weeks infants that survived received BMF on at least one day. As clinically expected, the oldest gestational age group (GA = 31 weeks) had the lowest use of HDM, BMF and PN, and the highest use of FM in comparison with all other GA groups except GA = 22 weeks (Table 1).

**Figure 1:**
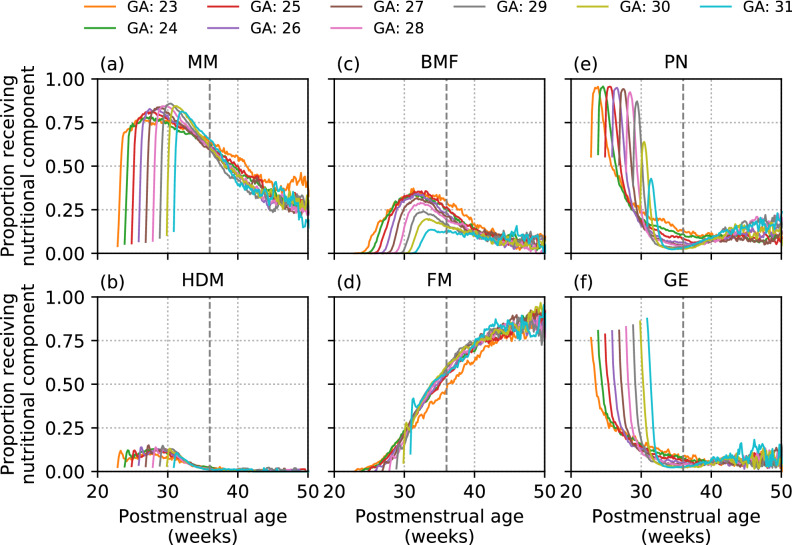
Time curves of the proportion of infants receiving a given nutritional component Time is expressed as postmenstrual age in weeks. Curves are stratified and colour-coded by infant gestational age groups (GA), computed on the set of infants still in care at a given postmenstrual age. The vertical grey dashed line indicates 36 weeks postmenstrual age. In (**a**–**f**) we show the proportion of infants who are still in care at a given postmenstrual age receiving each of the six components (MM: Maternal Milk, HDM: Human Donor Milk, BMF: Breast Milk Fortifier, FM: Formula Milk, PN: Parenteral Nutrition, GE: Glucose Electrolyte solution). We only considered GA > 22 weeks as, due to small sample size, values for GA = 22 weeks exhibited too much variation for meaningful interpretation.

The postmenstrual age at which a given nutritional component is administered was shown to vary across GA groups (Fig. 1). The proportions of all enteral components decrease over time prior to 36 weeks postmenstrual age, with the exception of FM, which increases.

### Stratification of the population by discovered nutritional patterns

**Figure 2:**
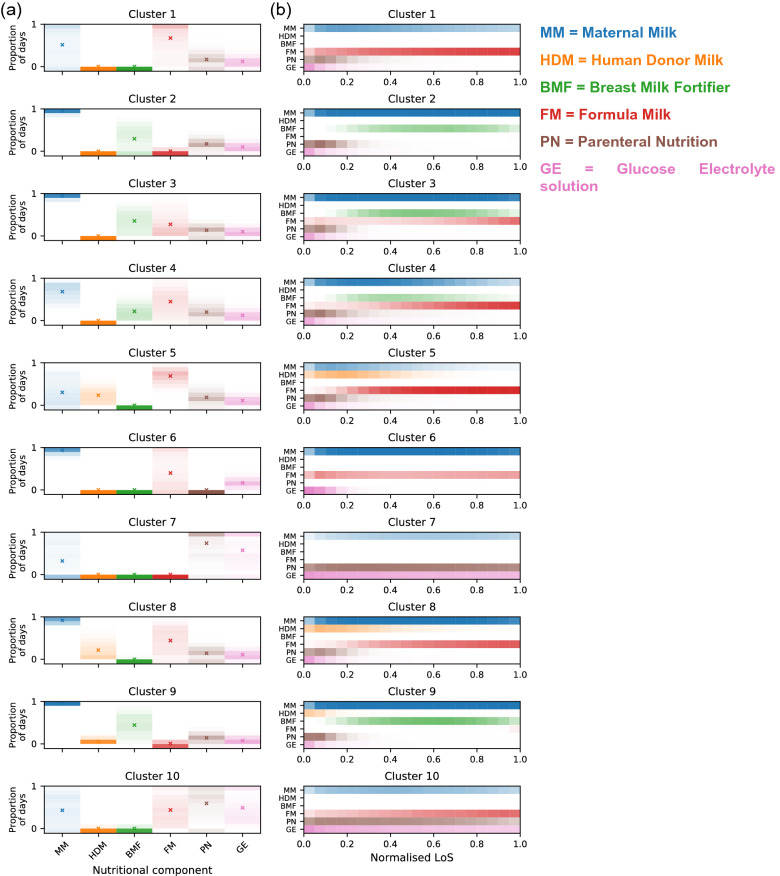
Composition of the 10 most common nutritional patterns ordered by size from largest to smallest. Nutritional patterns are encoded as the proportion of days over total time in care a nutritional component was given (0 to 1). (**a**) Histograms of proportion of infants receiving a nutritional component (one per column) over a given proportion of days. Shading = one-dimensional histograms (11 bins-width = 0.1-including 0). Colour intensity = density of infants fed with that proportion over time in care and is maximal if all infants fall into a single bin. Cross (“x”) = mean proportion of days averaged across all infants in the cluster. (**b**) Timeline of nutritional components administration over normalised LoS (20 equal bins), computed as average temporal sequences of nutritional events over time in care. Maximal bin value (and colour intensity) attained if all infants in the cluster received a particular nutritional component every day in that time bin.

The unsupervised clustering identified 46 nutritional patterns with distinct proportions of days for the administration of the six nutritional components (Table 2, Fig. 2), distinct temporal sequences of nutritional events (Fig. 2) and distinct characteristics on their population (nutritional clusters) (Table 2, Supplementary Table S1). The proportion of infants in each cluster is highly skewed, with 10 clusters covering 82% of the cohort (Table 1), and 24 clusters covering 95% of the cohort.

**Table 2:** Statistics on the within-cluster populations of the 10 most common nutritional patterns and the whole cohort.

Property type		Whole cohort	Cluster rank									
			1	2	3	4	5	6	7	8	9	10
Cluster size	N	45,679	11,052	5,471	4,868	3,951	2,935	2,913	2,707	1,382	1,207	758
	Proportion		24%	12%	11%	9%	6%	6%	6%	3%	3%	2%
	Cumulative proportion		24%	36%	47%	56%	62%	68%	74%	77%	80%	82%
Geographical location variables	North	30%	32%	27%	29%	36%	29%	27%	40%	14%	17%	26%
	Midlands	27%	28%	30%	33%	36%	18%	27%	27%	10%	15%	37%
	London	20%	19%	18%	17%	11%	22%	24%	16%	45%	36%	27%
	South	23%	22%	25%	20%	18%	30%	23%	17%	32%	32%	11%
Admission variables	Birth Year	2015	2015	2015	2015	2015	2015	2014	2015	2015	2015	2015
	Gestational Age (weeks)	29	30	29	29	28	29	31	25	30	28	28
	BW z-score	− 0.23	− 0.09	− 0.22	− 0.31	− 0.41	− 0.36	0.27	− 0.35	− 0.24	− 0.37	− 0.20
	Resuscitation	8%	6%	8%	7%	9%	9%	2%	17%	5%	9%	12%
	Antenatal Steroids	90%	89%	93%	93%	91%	89%	91%	85%	93%	94%	88%
	Sex (% girls)	46%	45%	46%	49%	47%	47%	43%	40%	47%	45%	40%
	Apgar 1 min	6	7	6	6	6	6	8	4	7	6	6
	Apgar 5 min	8	9	8	9	8	8	9	7	9	8	8
	Apgar 10 min	9	9	9	9	9	9	10	8	9	9	9
Outcome variables	Mortality	8%	1%	1%	1%	2%	1%	0%	76%	0%	0%	27%
	Necrotising Enterocolitis (NEC)	3%	2%	0%	0%	3%	2%	0%	11%	0%	0%	34%
	Bronchopulmonary Dysplasia (BPD)	27%	20%	26%	30%	46%	36%	2%	11%	15%	36%	40%
	Maternal Milk at Discharge	54%	34%	99%	90%	27%	8%	93%	32%	89%	98%	30%
	Length of Stay (days)	49	42	52	54	70	60	30	9	43	65	60
	W36 z− score	− 1.59	− 1.33	− 1.66	− 1.68	− 1.74	− 1.53	− 1.29	− 1.59	− 1.69	− 1.64	− 1.78
	W36dz	− 1.20	− 1.07	− 1.31	− 1.22	− 1.23	− 1.07	− 1.39	− 1.33	− 1.22	− 1.18	− 1.40
Nutritional variables	Maternal Milk	0.66	0.51	0.96	0.95	0.68	0.30	0.93	0.32	0.92	0.96	0.43
	Human Donor Milk	0.06	0.00	0.00	0.00	0.00	0.24	0.00	0.00	0.22	0.06	0.00
	Breast Milk Fortifier	0.14	0.00	0.30	0.35	0.22	0.00	0.00	0.00	0.00	0.44	0.00
	Formula Milk	0.38	0.67	0.00	0.27	0.45	0.69	0.40	0.00	0.44	0.01	0.44
	Parenteral Nutrition	0.21	0.17	0.18	0.13	0.20	0.19	0.00	0.74	0.14	0.14	0.59
	Glucose Electrolyte	0.17	0.12	0.10	0.10	0.12	0.11	0.17	0.57	0.11	0.08	0.49

Clusters are ranked according to population size (1 = largest cluster). All variables are reported as median values, except for proportions which are means. Proportions are reported as integer values, therefore 0% corresponds to < 0.5% and the sum across the geographical location variables may not be exactly 100%. Nutritional variables encode the nutritional patterns through the proportion of days a nutritional component was given over the total time in care (between 0 and 1).

The temporal nutritional sequences identified (Fig. 2) confirm the known practices of using PN immediately after birth, and the trade-off between MM and FM (e.g. cluster 1). However, nutritional patterns in the 3 largest clusters, covering over 47% of the population, show striking differences (Table 2). While 24% of infants received a trade-off between FM and MM over their neonatal stay (cluster 1), the other 23% (clusters 2–3) received MM on more than 95% of days, with BMF and FM introduced typically towards the end of stay in around half of cluster 3.

Cluster 43 (Supplementary Table S1) returned a clinically implausible nutritional pattern of high BMF use without other enteral nutrition. Subsequent investigation found this cluster contained n = 28 infants hospitalised in a single neonatal unit, consistent with erroneous data entry.

Comparing the average proportions of nutritional components in each of the 10 most common clusters against the population average, we observe that MM is greater in six clusters, HDM in three clusters, BMF in four clusters, FM in six clusters, and PN and GE in the same two clusters. Combining observations from Table 2 and Fig. 2, we summarize the nutritional clusters as follows:

**Cluster 1** (n = 24%; N = 11,052; median GA = 30 weeks): Trade-off between MM and FM and minimal BMF and HDM.

**Cluster 2** (n = 12%; N = 5,471; median GA = 29 weeks): Large amounts of MM, with some BMF and minimal FM and HDM.

**Cluster 3** (n = 11%; N = 4,868; median GA = 29 weeks): Similar to cluster 2, except for some use of FM, typically towards the end of time in care.

**Cluster 4** (n = 9%; N = 3,951; median GA = 28 weeks): Large amounts of PN, with FM typically introduced early during time in care, and minimal use of HDM.

**Cluster 5** (n = 6%; N = 2,935; median GA = 29 weeks): Early feeding with HDM, low use of MM, and minimal BMF.

**Cluster 6** (n = 6%; N = 2,913; median GA = 31 weeks): Minimal PN, some GE, and FM more prevalent than MM.

**Cluster 7** (n = 6%; N = 2,707; median GA = 25 weeks): Prolonged PN and GE, combined with a low proportion of MM.

**Cluster 8** (n = 3%; N = 1,382; median GA = 30 weeks): Similar to cluster 5, with HDM typically introduced early during time in care but with a higher proportion of MM.

**Cluster 9** (n = 3%; N = 1,207; median GA = 28 weeks): Similar to clusters 2 and 3, with a large proportion of days involving MM and BMF, but additionally with HDM introduced early during time in care.

**Cluster 10** (n = 2%; N = 758; median GA = 28 weeks): Similar to cluster 7 but FM used in addition to MM.

We return to examine the relationship between nutritional cluster, geographical location and outcomes later.

### Reproducibility of the stratification

#### Reproducibility over multiple runs of the unsupervised DPGMM clustering algorithm on the whole cohort

Twenty independent runs of our model fit on the whole cohort (n = 45,679 infants), led to the discovery of 43 to 51 nutritional clusters (Supplementary Table S2). The 46 nutritional clusters evaluated in our results were selected from a run corresponding to an “intermediate” result.

Reproducibility of the discovered clusters was quantified using the F-measure^35^. Initially designed to compare a proposed clustering of samples to a ground-truth stratification into classes, the F-measure is computed as a weighted average over all clusters of two metrics: cluster purity (i.e. proportion of the predominant class label within a cluster) and cluster completeness (i.e. proportion of the samples from the predominant class label that are included in the cluster). It takes values between 0 (total disagreement) and 1 (perfect agreement) between the proposed clustering and the ground-truth class labels. To examine the reproducibility of the stratifications across the 20 runs, we arranged them in 10 pairs, randomly taking one clustering as the ground-truth class labels, and we obtained a mean value of F_total_ = 0.82 ± 0.01 (Supplementary Table S2). Coverage of the population from the 10 dominant clusters was very stable (mean 81% ± 1%), and agreement for the clusters covering 80% of infants was very high (F_total_ = 0.85 ± 0.02). Qualitative comparison of nutrient proportions (Supplementary Fig. S1) showed very high reproducibility in the largest clusters, while the smallest (rarer) ones showed some within-cluster variability mostly within PN components.

#### Reproducibility over training on sub-cohorts

We constructed two sub-cohorts by separating the infants born in odd/even years (infants born in 2018 were excluded due to only half the year being included in the whole cohort). These two sub-cohorts showed no significant differences for admission, geographical location and outcome variables, as measured by Fisher’s Exact and Mann–Whitney U statistical tests (Supplementary Table S3). Alternatively using the odd/even birth year sub-cohorts as training/validation sets for our stratification (clustering) method, we checked that our generative model learned on training data can generate synthetic samples statistically similar to the held-out validation data and is therefore representative. To do this we performed a posterior predictive check^36^ which measures the probability (*p* value) of a test statistic (e.g. population mean, variance, likelihood) for the synthetic data compared to the validation data. Average *p* value over 40 repetitions for the likelihood test statistic was 0.56 ± 0.01, indicative of good generative capability.

Regarding reproducibility of assigning individual infants to nutritional clusters (i.e. stratification), utilizing again the F-measure, we found a mean value of F_total_ = 0.78 for repeated model fits on the same data (two runs on the same sub-cohort), and F_total_ = 0.76 for held-out data (a model fitted on the odd birth years and applied to stratify the even birth years sub-cohort and vice versa). This demonstrates 1) reproducibility of our model over multiple runs while reducing the training cohort size by a half and 2) reproducibility of clustering of held-out data when using two statistically similar sub-cohorts.

Further, pairing clusters obtained from clustering the odd/even birth year sub-cohorts separately (using the maximal F-measure for each pair), we found 94% of admission and outcome variables to have a non-significant difference (*p* value > 0.05 with Bonferroni correction, across 555 tests), which indicates that our proposed stratification into nutritional clusters did not generate significantly different aggregates over independent variables, when trained on statistically similar sub-cohorts.

## Discovering associations between nutritional practices, outcomes and geographical location

**Figure 3:**
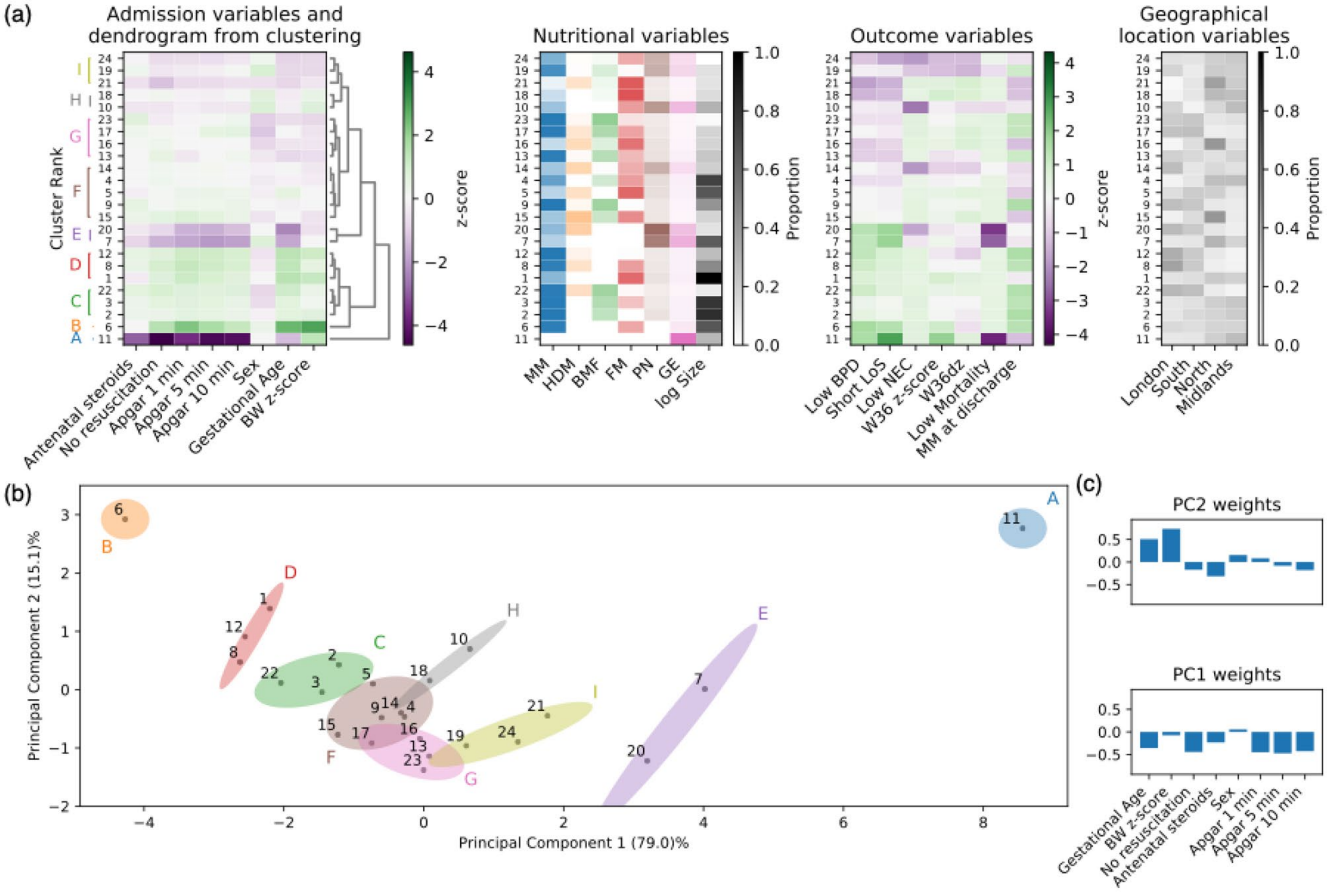
Aggregation of nutritional clusters based on admission variables identifies 9 admission groups to further assess association with geographical location and outcome variables. Hierarchical clustering and PCA performed on the 24 most populated nutritional clusters (covering 95% of the whole cohort) using admission variables (shown in (**a**)) to characterize infants in each cluster. Nutritional clusters indexed according to size rank. The 9 admission groups, defined by visual inspection, are encoded with letters A to I on the hierarchical clustering dendrogram (**a**) and on the PCA projection plot (**b**). (**a**) Admission z-scores values (used for clustering), along with outcome variables (z-scores), nutritional and geographical location variables (mean proportions, Table 2). Colour coding for admission and outcome variables uses green for “favourable” and purple for “unfavourable” values (e.g. a high z-score value is unfavourable for NEC while favourable for MM at discharge). Feature columns in admission, outcome and geographical location variables are also reordered based upon hierarchical clustering. (**b**) Projection of the nutritional clusters (plotted as points with cluster index as in (**a**)) along the two main principal components returned by PCA (PC1 and PC2, explaining 94% of the variance). Admission groups A to I from (**a**) are reproduced on the PCA plot with ellipses whose axes are based on the covariances of the group’s coordinates along PC1 and PC2. (**c**) Contributions of individual admission variables to the first two principal components (PC1 and PC2). PC1 is driven by gestational age, resuscitation and Apgar scores, while PC2 is driven by gestational age and birth weight z-score.

To take further advantage of the cohort stratification into nutritional patterns, we attempted to minimize the influence of admission characteristics before examining associations with geographical locations and outcomes. To do this, we performed hierarchical clustering and principal component analysis (PCA) on the admission variables of the 24 most populated nutritional clusters that cover 95% of the population (Table 2 and Supplementary Table S1). Admission and outcome variables (Fig. 3a) were defined by transforming cluster means as z-scores to encode values as above or below whole-clusters “average”.

The hierarchical clustering dendrogram (Fig. 3a) and the PCA projection (Fig. 3b,c) are in high agreement regarding nutritional clusters deemed “similar” according to their admission variables. Based on visual inspection, we identified 9 distinct admission groups (of variable sizes) of nutritional clusters.

We now further discuss some admission groups of specific clinical value. **Admission groups A** and **B** are two outliers on the PCA projection plot (Fig. 3b), composed of two isolated nutritional clusters (respectively cluster 11 and 6), of relatively large size (respectively 2% and 6% of whole-cohort size) and with respectively unfavourable and favourable admission variables. These clusters are also ones with highest and lowest mortality rates among all clusters (95% and 0% respectively). Based on these observations, these nutritional clusters appear too different from the other ones to further investigate cluster-based influence of nutritional patterns.

Regarding potential associations between nutritional patterns and geographical location, we identified two admission groups of note: 1) **Admission group C** (clusters 2–3, 22) includes above-average gestational age infants (GA = 29–30 weeks versus 29 weeks in the whole cohort), with high survival (mortality < 3%) and low prevalence of NEC (< 1%). Clusters 2–3 are large, reflecting widely used nutritional practices, and differ only in the use of FM, a well-known source of variation in infant nutrition. The small cluster 22 shows additional use of HDM and is more frequent in London and South geographical locations (compared to whole cohort average). This admission group indicates an opportunity to further investigate geographical factors in nutritional practices related to use of HDM. 2) **Admission group D** (clusters 1, 8, 12) includes above-average gestational age infants (GA = 30 weeks versus 29 weeks in the whole cohort), with favourable admission variables, high survival (mortality < 2%) and low prevalence of NEC (< 3%). Cluster 1 is the largest, with minimal geographical location bias, but with low MM at discharge (34% versus 54% whole cohort). In comparison, clusters (8, 12) have high MM at discharge (89%, 99%), and are more frequent in London and South geographical locations (compared to whole cohort average). Furthermore, comparing clusters 12 versus 8, cluster 12 has less use of HDM (8% versus 27%) and minimal use of FM (1% versus 44%). These observations indicate some opportunity to identify geographical factors in nutritional practices and associated influence on MM at discharge.

Regarding the previously identified six nutritional clusters with high NEC prevalence, and where increased use of PN was confirmed by clustering, clusters (7, 20) and (19, 24) fall within similar admission groups (E and I respectively). NEC is a rare condition, hence challenging to study using conventional approaches such as randomised controlled trials. These admission groups could be used in future work to eliminate admission variables as drivers, and identify hypotheses relating to the effect of differences in nutritional practice on likelihood of NEC.

Regarding the previously identified 15 nutritional clusters with high BPD prevalence, they predominantly aggregate in admission groups F, G, H and I and have several identifiably different nutritional patterns. Among those, in group F, cluster 9 is more frequent in London relative to clusters 4 and 16. These admission groups could be used to eliminate admission variables as drivers, and identify hypotheses relating to the effect of differences in geographical location and/or nutritional practice on likelihood of BPD in future work.

## Exploring the association between nutritional regimen and weight gain and BPD

We further considered two scenarios to identify associations between nutritional practice and outcomes using a recently introduced methodology to reduce the effect of confounders^33,34,37,38^. We selected infants with similar admission variables (Fig. 3b), still in care at 36 weeks postmenstrual age, and alive at discharge.

As a first scenario, we verified if we could confirm the well-recognised relationship between the use of FM and higher weight gain modelled using the weight z-score at 36 weeks postmenstrual age (W36 z-score). We used nutritional clusters 13 and 16, which are in the same admission group G (Fig. 3b) but have distinct W36 z-score values (− 1.87 versus − 1.53, and − 1.59 average for the whole cohort). Linear regression returned an adjusted r^2^ = 0.46, showing that nearly half of the variation is explained by the model. Our results (Table 3a) identified “Birth weight z-score” (BW z-score) as a significant covariate (coefficient = 0.72; *p* value < 0.001), indicating that an infant born with BW z-score value of 1 unit more will have a W36 z-score value greater by 0.72. It also showed a significant effect of the nutritional pattern (coefficient = −0.33; *p* value < 0.001) suggesting that belonging to cluster 16 relative to cluster 13, independently contributes a difference of 0.33 to the W36 z-score. From the nutritional patterns of these clusters we can further infer that more FM, along with less MM and less HDM is associated with higher weight gain, in keeping with well-established clinical knowledge^7^.

**Table 3:** Association between nutritional patterns and 2 clinical outcomes.

	Coefficient	95% Confidence Interval	*p* value
**(a)**			
Intercept	**− 1.68**	**[− 1.74, − 1.62]**	**< 0.001**
BW z-score	*0.72*	*[0.64, 0.79]*	< *0.001*
Antenatal Steroids	0.15	[− 0.07, 0.37]	0.185
Apgar 1 min	0.00	[− 0.04, 0.05]	0.861
IMD decile	− 0.01	[− 0.04, 0.02]	0.588
Smoking in Pregnancy	0.05	[− 0.09, 0.19]	0.504
Sex	− 0.06	[− 0.18, 0.06]	0.301
Gestational Age	*0.06*	*[0.01, 0.11]*	*0.017*
Midlands and east	− 0.09	[− 0.27, 0.1]	0.364
North	− 0.09	[− 0.27, 0.09]	0.306
South	0.02	[− 0.16, 0.21]	0.796
Nutritional Treatment	**− 0.33**	**[− 0.46, − 0.2]**	**< 0.001**
Z_1_	0.03	[− 0.09, 0.15]	0.641
Z_2_	− 0.04	[− 0.16, 0.07]	0.482
Z_3_	0.00	[− 0.1, 0.11]	0.940
**(b)**			
Intercept	− 0.15	[− 0.36, 0.05]	0.143
BW z-score	*− 0.64*	*[− 0.92, − 0.37]*	< *0.001*
Antenatal Steroids	0.27	[− 0.49, 1.03]	0.491
Apgar 1 min	− 0.09	[− 0.24, 0.07]	0.263
IMD decile	0.00	[− 0.15, 0.15]	0.977
Smoking in Pregnancy	0.05	[− 0.48, 0.58]	0.862
Sex	*− 0.58*	*[− 1, − 0.15]*	*0.008*
Gestational Age	*− 0.43*	*[− 0.58, − 0.28]*	< *0.001*
Midlands and east	0.02	[− 0.62, 0.66]	0.941
North	0.38	[− 0.27, 1.02]	0.254
South	0.42	[− 0.19, 1.04]	0.176
Nutritional Treatment	*− 1.16*	*[− 1.67, − 0.65]*	< *0.001*
Z_1_	− 0.26	[− 0.66, 0.15]	0.216
Z_2_	0.21	[− 0.19, 0.61]	0.309
Z_3_	− 0.10	[− 0.54, 0.34]	0.648

Regression coefficients, 95% confidence intervals and *p* values are reported for all tested covariates, and the three latent variables (Z_1_, Z_2_ and Z_3_) of the factor model that facilitate deconfounding. We excluded infants with any missing values for the studied variables and used London as the reference category for geographical location. (a) Linear regression on weight z-score at 36 weeks postmenstrual age (W36 z-score), comparing clusters 13 (n = 371) and cluster 16 (n = 339). (b) Logistic regression on BPD, comparing cluster 17 (n = 376) to 18 (n = 283). Coefficients in (a) measure effect on the outcome by a change of one unit in the corresponding covariate. Coefficients in (b) measure change in log-odds ratio of the outcome by a change of one unit in the corresponding covariate. “Nutritional Treatment” = binary covariate encoding inclusion in one cluster versus the other. Significant (*p* < 0.05) covariates are highlighted in bold/italic if inducing a negative/positive effect on the outcome variable when the associated covariate is quantitatively positive.

As a second scenario, we considered the relationship between nutritional patterns and BPD. We used nutritional clusters 17 and 18 with similar admission variables, low NEC prevalence and very different BPD prevalence (36% versus 63%, and 27% average for the whole cohort). Logistic regression returned a pseudo r^2^ = 0.23, illustrating a reasonable model fit. Our results (Table 3b) indicate a significant effect of nutritional pattern (coefficient = −1.16; *p* value < 0.001) suggesting that belonging to cluster 17 is related to decreased BPD. From inspecting the nutritional pattern of cluster 17, we observe a greater proportion of MM, HDM and BMF, while cluster 18 has a greater proportion of FM. These results support the hypothesis that human milk may be protective against BPD^12^. Geographical location was not identified as significant suggesting that the effect of the nutritional pattern on both W36 and BPD is independent of the regional bias observed between the compared clusters.

## Discussion

In this study, we deployed, for the first time, an agnostic generative unsupervised machine learning approach, to describe and explore patterns of nutritional practice in very and extremely preterm infants across the entire population of admissions to neonatal units in England over a six-year period. We used our approach to stratify the population based upon nutritional components alone, without prior knowledge of clinical practice or variables relating to admission, discharge or geographical location. Using this agnostic approach, we identified well-recognised common nutritional practices, as well as less common approaches. We were able to relate nutritional practices to infant admission profiles. We also identified previously known, as well as novel, associations between nutrition and outcomes. Our study provides a framework for data-driven approaches to investigate and generate new hypotheses relating to variation in clinical practice and the impact on clinical outcomes.

Firstly, independent of clustering, we described the delivery of nutritional components to the cohort, and variation by gestational age. This allowed the nature, timing and extent of each nutritional component to be considered. We showed clinically plausible patterns, such as early use of PN and greater use of BMF among low gestational age infants. We also found, as anticipated, that the most mature infants had lower use of HDM, BMF and PN.

Secondly, the importance of identifying common, reproducible nutritional patterns as clusters is supported by the high number of theoretically possible combinations (around 13.8 billion combinations if considering 6 distinct binary nutritional components over a median length of stay of 49 days). We encoded nutritional patterns as the proportion of days each component was given over the entire length of stay and identified 46 clusters of nutritional practice. Motivation for using DPGMM as the unsupervised clustering method came from the desire to provide more precise and adaptable clustering than the scalable K-means and to be tractable across the whole population so as to not require a classifier for predicting cluster membership from fitting to a smaller subset, as required in other similar studies^27^. An advantage in utilising a Bayesian approach is that a predictive distribution is freely available, naturally accounting for uncertainty in parameterisations through the approximation of the full posterior distribution. The relatively large number of discovered nutritional patterns (n = 46) illustrates the considerable variation in clinical practice. This notwithstanding, 10 large nutritional clusters explain nutritional practice for 82% of the population. The NNRD is a source of “real-world” data (data derived from real-world as opposed to experimental settings) that undergoes multiple levels of quality assurance. This notwithstanding, we identified a cluster of infants from a single neonatal unit in which the use of BMF was documented without any other enteral nutrition. This is clinically implausible, highly suggestive of a data recording error, highlighting the additional utility of the unsupervised clustering approach for data quality assurance.

In unsupervised machine learning procedures such as this, there is no “true” number of nutritional clusters. By using a Dirichlet process we let the data dictate the number of nutritional patterns rather than following the common practice of constraining the number *a priori*. Our results regarding reproducibility confirmed that the exact number of nutritional patterns does not matter, as variability only concerns small nutritional clusters with few infants. The largest nutritional patterns have high reproducibility in terms of nutritional practices and within-cluster population characteristics.

Thirdly, the nutritional clusters showed that nutritional practices differ with respect to geographical location. Understanding the drivers of locational variation is an important area for future exploration. We confirmed clinical plausibility by identifying known, well-recognised relationships such as a higher proportion of PN days in infants with high mortality or NEC, lower PN in infants with more favourable admission characteristics and outcomes, and the effect of FM on greater weight gain. We extended our analysis to identify a potentially positive impact of MM in reducing the risk of BPD, a matter of great interest clinically. In a recent meta-analysis^12^ involving predominantly observational data, Huang et al.suggested that MM might reduce the likelihood of BPD. However, this is difficult to put to the stringent test of randomisation as it would be unethical to withhold MM from preterm infants. Our results therefore provide tentative independent support for this hypothesis.

The strengths of our study include the use of a generative model with approximate inference making full use of a Bayesian formulation to model the posterior distribution of multivariate Gaussians to describe the population. This is in contrast to alternative approaches such as K-means or hierarchical clustering, which require the design of a companion classification strategy such as Nearest Centroid^27^. In addition, the generative model allowed us to perform posterior predictive checking as a rigorous means for assessing the model’s representation of reality and its utility. Simple approaches such as K-means also struggle to capture complex heterogeneities in the data due to the nature of the clustering optimisation which treats feature space as isotropic when identifying patterns and therefore fails to identify covariances such as the anticorrelation between the use of MM and FM. A further strength of our study was the use of a complete, whole population dataset containing records on every baby admitted for NHS neonatal care in England over the defined time period. This eliminates a major limitation of many observational and randomised studies, namely inclusion bias and lack of generalisability. Allowing a flexible number of patterns to be learned and letting the data determine the number and cluster assignment leads to the advantageous discovery of rare clusters populated with only few tens to hundreds of infants. These clusters provide opportunity to identify unusual but potentially informative patterns and outliers. The deconfounding framework utilised has two advantages for exploring associations: it is readily applicable and uses a testable model fitting assumption that, if met, eliminates the possibility of unknown multi-cause confounders (unobserved confounders which affect more than one of the variables considered to be potential causes).

This study has limitations that warrant consideration. These relate to our selection of observed variables, the use of feeding patterns as representative of “treatment” and the extent to which we can reliably make causal inferences from the identified associations, a significant challenge for all analyses using observational data. Regarding the selection of observed variables, we did not use the variable hospital of care, although it is a possible baseline determinant influencing both feeding and care practices. Our decision was based on the fact that infants often move between neonatal units depending on their care needs (approximately 35% of babies moved in this study), hence relating an outcome to a specific neonatal unit would be inappropriate. We therefore only used region of care variables in our analyses.

Regarding the use of feeding patterns as representative of “treatment” we acknowledge that, from an epidemiologic point of view, these could be considered “mediating variables” that transmit the effect of an antecedent (e.g. geographical location) to a dependent variable (e.g. the outcome)^39^. Alternatively the strong likelihood of multiple interactions between nutritional components could be construed as justification for our approach. This topic merits further investigation.

We consider our findings associations, and not evidence of causality, but used a principled machine learning approach to reduce the presence of confounding. This could be strengthened in the future through the use of more sophisticated predictive models, alternative factor model specification, incorporation of additional covariates, and parallel use of other methods to elicit causal inferences, such as propensity score matching or instrumental variable analysis^40,41^. Considerations around the timing of care practices could also be utilised given the rich daily temporal records available in the NNRD.

In summary, our study demonstrates the potential of unsupervised machine learning applied to real-world data to provide interpretable insights into variations in clinical care and their potential impact. Data-driven discovery allows identification of variation within groups of infants with similar background characteristics. Patient care could theoretically benefit from structured exploration through for example, combining detection of non-random associations to identify possible disease determinants, and predictive modelling to uncover patient pathways and interventions that might alter outcomes, especially in situations not amenable to randomised evaluation. Future utility of machine learning on healthcare data will be enhanced by ensuring, as done in our work, that models are reproducible, simple to understand and manipulate, and efficient at generating evidence at a faster pace and lower cost than conventional approaches.

## Materials and methods

### Study design

We used the National Neonatal Research Database (NNRD)^42^, a mature ongoing longitudinal registry that commenced in 2007 and that contains detailed clinical information extracted from the electronic patient records of all admissions to neonatal units in England, Wales and Scotland^43^. Out of nearly one million infants and 12 million daily records of care, we selected all very and extremely preterm infants, defined as born below a gestational age of 32 weeks. To consider only the period of time for which there was full coverage across all neonatal units in England (after 2012), and infants who had completed their time in care, we further restricted the birth year range from January 2012 to July 2018. To study region-specific trends, we further excluded infants who received care in more than a single region during their length of stay (Supplementary Fig. S2). For logical consistency, episodic data and daily summary data needed to be converted into a single entry per infant, involving linking records from different neonatal units. Missing data was present in both episodic and daily summary data (Supplementary Fig. S2). For episodic data, to obtain the appropriate value for variables considered constant, we selected the last non-missing value (Last Observation Carried Forward approach) where the episodes were ordered by anonymised admission time across episodes. For daily summary data, the curation process reverts all entries to midnight of the day of data entry. We reset the clock of each infant’s trajectory (the multivariate time series of variables across the entire length of stay) to zero for the first entry and considered the time from birth to this entry to calculate the age in days for that infant. We removed all day entries where there were any missing data in the variables of interest. Entries marked with the same timestamp were merged by taking the union of events that occurred between the set of entries with matching timestamps. Such instances occur when an infant is transferred to a different neonatal unit and records may be entered at each unit during that day. With this procedure complete, we selected a single complete entry per day of nutritional events for n = 45,679 infants.

Weight z-scores were calculated using the LMS method of Cole and Green^44^ using sex, gestational age, postnatal age and birth weight^45,46^.

We divided the variables of interest into four categories: (1) admission, (2) nutritional, (3) outcome, (4) neonatal unit geographical location.

**Admission variables**: We considered the following variables as the primary representation of infant admission characteristics:

- Birth year (integer): the integer year in which the infant was born.
- Gestational age (GA; integer): the number of completed weeks of gestation at birth.
- Birth weight z-score (BW z-score; continuous): the birth weight of the infant adjusted for sex and gestational age to produce a z-score.
- Resuscitation (binary): defined as 1 if cardiac compression and/or resuscitation drugs were used at birth, 0 otherwise.
- Antenatal steroids (binary): defined as 1 if any administration of steroids to the mother prior to birth, 0 otherwise.
- Sex (binary): defined as 0 for boys and 1 for girls (arbitrary dummy coding where the mean of this variable over infants returns the proportion of females).
- Apgar scores at 1, 5 and 10 min (ordinal): summary health score between 0 and 10 over five categories recorded for the infant at each indicated time from birth.

**Additional admission variables:**

- Index of Multiple Deprivation score (IMD decile; integer): the decile measuring the deprivation score for the mother’s location of residence at the time of the infant’s birth, with 1 being the most deprived and 10 being the least deprived^47^.
- Smoking in Pregnancy (binary): defined as 1 if mother confirmed smoking during pregnancy, 0 otherwise.

**Nutritional variables:** For each infant, nutrition is recorded in the NNRD daily summary tables using six variables. For the enteral component these are Maternal Milk (MM), pasteurised Human Donor Milk (HDM), Breast Milk Fortifier (BMF) and Formula milk (FM) and for the parenteral component, Parenteral Nutrition (PN) and Glucose Electrolyte solution (GE). Each variable is recorded daily as having occurred or not (binary event). If any of these nutritional events was missing in a single daily record, that record is excluded. We converted the daily record of these six variables into a single vector per infant by counting the number of days on which each nutritional variable was given and dividing this by the length of stay. By doing so, each variable is encoded as a number between 0 and 1 representing the proportion of days an infant received this nutritional component.

**Outcome variables:** We considered the following seven outcomes, informed by those identified as important by parents, patients, clinicians and researchers^48^, to define the outcome variables of each infant at discharge from neonatal care:

- Mortality (binary): defined as 1 if death is entered as the discharge destination for the last non-missing episode of care ordered by discharge time, 0 otherwise.
- Necrotising enterocolitis (NEC; binary): defined as 1 if severe NEC occurred, 0 otherwise. As milder forms of necrotising enterocolitis (NEC) are difficult to identify consistently and lack an agreed case-definition, we restricted ourselves to evaluating *severe* NEC. In accordance with the definition developed by Battersby et al*.*^10^, severe NEC is defined as NEC that required surgery or was confirmed at death (see Supplementary Table S4 for how severe NEC is measured in the NNRD). NEC influences nutritional practice as treatment involves a period (commonly 7–14 days) without enteral nutrition and with PN or GE. Infants with NEC may also have difficulty in re-establishing enteral nutrition, hence requiring prolonged periods of PN.
- Bronchopulmonary dysplasia (BPD; binary): defined as 1 if BPD occurred, 0 otherwise. BPD is defined as receiving any respiratory support or supplemental oxygen at a postmenstrual age of 36 weeks. Set to NA for infants who died prior to 36 weeks postmenstrual age.
- Maternal milk at discharge (binary): defined as 1 if the infant received some MM on either of the last two days of time in care, 0 otherwise.
- Length of Stay (LoS; integer): the difference (in days) between the maximum time and minimum time from the daily summary table, representing an infant’s total time in care.
- Weight z-score at 36 weeks postmenstrual age (W36 z-score; continuous): weight at a postmenstrual age of 36 weeks converted to a z-score. Set to NA for infants who died prior to 36 weeks postmenstrual age.
- Change in weight z-score from birth to 36 weeks postmenstrual age (W36dz; continuous): the difference between W36 z-score and BW z-score. Set to NA for infants who died prior to 36 weeks postmenstrual age.

**Geographical location:** binary mutually exclusive variables for four regions (North, Midlands, London, South). The value is set to 1 for the region of the host neonatal care unit and 0 for the three other regions. We used the following grouping of regions to define geographical locations as entered in the NNRD:

- **North**: Cheshire, Lancashire, Cumbria, Northumberland, Durham, Tyne & Wear, and Yorkshire.
- **Midlands**: Leicestershire, Lincolnshire, Essex, Hertfordshire, Bedfordshire, Cambridgeshire, Norfolk, Suffolk, Worcestershire, Birmingham, Warwickshire, Staffordshire, Shropshire and Nottinghamshire.
- **London**: South London, West London, North East and Central London.
- **South**: Kent, Surrey, Sussex, Cornwall, Somerset & Avon, Devon, Buckinghamshire, Berkshire and Dorset.

### Model design for unsupervised clustering

We applied unsupervised clustering to the nutritional components of the whole cohort aggregated in a N × L nutritional matrix X_nl_, where each row corresponds to an infant and each column corresponds to a nutritional variable. During the clustering process, infants are grouped on the basis of having received a similar nutritional regimen during their neonatal unit stay. We intentionally excluded any other characteristics (geographical location, admission or outcome).

We chose the Dirichlet Process Gaussian Mixture Model (DPGMM) to perform clustering^31,32^. A GMM is a generative model that fits a mixture of Gaussian distributions to the observed variables. Each cluster is represented by a Gaussian distribution parameterised by a vector of mean values and a covariance matrix describing correlations between variables. The “Bayesian” aspect of the model incorporates a prior on the mean and covariance of the clusters and learns an approximate posterior over these parameters. The priors for the cluster’s mean and covariance are centred on the mean and covariance of the entire dataset respectively. Using a “Dirichlet Process” for the fitting procedure (in our case the posterior inference) optimizes the number of clusters to use through the manipulation of an extra parameter called the weight concentration. This parameter controls whether large numbers of evenly sized or small numbers of unevenly sized clusters better fit the data. With the approximate Bayesian posterior inference, the model allows uncertainty in the estimated cluster shapes and assignments to be taken into account. The output of the DPGMM algorithm returns a generative model, a set of nutritional patterns, and probabilities for each infant as having each pattern. We defined our final nutritional clusters by assigning each infant to the pattern with the highest probability.

The DPGMM has several advantages over simpler unsupervised clustering approaches such as K-means. Firstly, the Dirichlet Process estimates the optimal number of clusters as part of the posterior inference procedure whereas K-means requires an *a priori* estimate of the number of clusters to be identified. Secondly, using Gaussian distributions assigns means and covariances between variables that can be straightforwardly interpreted. It also enables complex covariance patterns between variables such as non-isotropy and anti-correlations, while K-means is limited to isotropic covariance. Thirdly, as a generative model, it enables generation of synthetic data and rigorous statistical interrogation of the quality of the model. Lastly, the Bayesian posterior implicitly allows overlap between clusters and captures uncertainty of a sample’s cluster assignment, making the approach robust to outliers while K-means favors large inter-cluster distances and is very sensitive to outliers.

The approximate inference involves initialisation with random parameter values, and joint optimization of the number of clusters and their Gaussian distributions maximizing an objective called the evidence lower bound (ELBO) that measures the quality of the approximate model fit. We stopped iterations when ELBO improvement fell below a tolerance threshold. We set the tolerance to 0.001 but found our results to be insensitive to this parameter. A “weight-concentration” parameter for the Dirichlet process controls our prior expectation for a small or large number of clusters. We set this parameter to 10^–5^ but found our results to be insensitive to this parameter in the range [10^–5^, 10^5^]. All analyses were performed using *Scikit-Learn*^49^.

### Model Evaluation

**Reproducibility:** Since there are no ground-truth clusters (i.e. the composition of nutritional patterns and assignments of infants to clusters are not known *a priori*), we performed three surrogate validation tests, as commonly done in unsupervised clustering^50^.

First, we measured reproducibility over repeated DPGMM model fits on the whole cohort using the F-measure^35^ to quantify clustering agreement between two runs. Using 20 runs with different random seed initialisations, we performed 10 comparisons between distinct pairs.

Second, we measured reproducibility over sub-populations by splitting the whole cohort into a training and validation set^50^. Here we split the infants into odd (2013, 2015, 2017) and even (2012, 2014, 2016) birth years (excluding infants born in year 2018 as only half of the year is included in the cohort). Treating one cluster assignment as “true” (model fitted on the training set) and the other one as “predicted” (model fitted on the validation set), the infants with odd/even birth years are alternatively utilised in the roles of training or validation sets. We measured clustering agreement with the F-measure, and the ability for the fitted models to predict cluster assignment using posterior predictive check^36^, providing a measure of the quality of model fit.

**Clinical plausibility:** To evaluate the clinical plausibility of the discovered nutritional patterns, we compared the nutrient proportions of the top 10 largest clusters and assessed whether these were clinically interpretable. We also compared admission and outcome variables between nutritional clusters using Mann–Whitney U tests on variables derived from continuous entries and Fisher’s Exact Test on variables derived from binary entries^16^.

**Clinical utility:** To assess clinical utility, we grouped nutritional clusters according to the primary admission variables of the infants. We computed the mean admission variables within each individual nutritional cluster and then standardised these values as z-scores. The z-score for a given variable for a given cluster was calculated by subtracting the mean across all clusters and normalising by the standard deviation across all clusters for that variable. We then performed hierarchical clustering of nutritional clusters^51^ using Euclidean distance and the Ward’s method to minimise overall within-cluster variance during agglomeration. We also applied principal component analysis (PCA) to the cluster z-scores to create a two-dimensional projection for interpreting and visualising their similarity.

### Measuring associations of nutritional patterns with outcomes

To measure associations between nutritional patterns and given outcomes, we followed a method previously applied to electronic health records^33,34,37,38^ to adjust for confounding factors (observed and unobserved) that could affect both treatment and outcome. This recent theory introduces the potential to reduce confounding effects by fitting a model that infers latent confounders from observed variables making additional unknown multi-cause confounders theoretically not possible. Alongside its potential for reducing confounding, the ability to test the fitted model, and therefore have testable modelling assumptions underlying the inference, is a strength of the method. However, the approach has been subject to some debate^38^ and we proceed cautiously here without specific causal claims. We considered nutritional patterns as “treatments” and as potentially confounding covariates seven admission variables and a geographical location variable with three categories (Midlands, North and South) versus London as the reference region. Admission variables include five already used to describe the nutritional clusters (BW z-score, Antenatal steroids, Sex, GA, Apgar 1 min) and two additional ones (IMD decile, Smoking in Pregnancy). “Apgar 5 min”, “Apgar 10 min” and “Resuscitation” variables were excluded to avoid collinear admission variables that can affect inferences with the deconfounder method. “IMD decile” and “Smoking in Pregnancy” were added to reduce the possibility of unknown single-cause confounders relating to nutritional pattern and outcome alone. We performed modelling on infants still in care at 36 weeks postmenstrual age, alive at discharge, and without any missing values for the variables under consideration.

Following the approach introduced by Wang and Blei^33^, we applied the deconfounding method on pairs of nutritional clusters C_1_ and C_0_, with the “nutritional treatment” variable encoding the treatment under evaluation and set as a binary variable equal to 1 for infants in cluster C_1_ and 0 for infants in cluster C_0_. We first mean-centred all covariates (for ease of coefficient interpretation). We then modelled the joint distribution of the input covariates with a probabilistic factor model that introduces latent variables to eliminate the possibility of any unobserved multi-cause confounders. Using probabilistic PCA^52^, with three latent variables, we trained a factor model on 80% of the studied cohort, and verified adequacy of the fit using the *p* value of the posterior predictive check on the 20% held-out data against simulated data from the model fit. A *p* value close to 0.5 is required to confirm that the factor model is able to adequately describe the joint distribution of all covariates, which we ensured was the case in our two model fits.

Utilising the mean latent variables of the fitted factor model as additional covariates, we then regressed against the outcome of interest, using linear/logistic models for a continuous/binary outcome. We measured the quality of fit with standard metrics: adjusted-r^2^ in linear regression and pseudo-r^2^ in logistic regression. Regression models were fitted using the Python package *statsmodels*^53^. Regression coefficients, 95% confidence intervals and *p* values for all considered covariates are returned. A *p* value < 0.05 was considered significant and the value/sign of a coefficient indicate the size/direction of the covariate’s effect. As the covariates are mean-centred, fitted coefficients of the regression show the size of the effect in the units of the covariates.

### Ethical approval

The National Research Ethics Service has approved the NNRD as a research database (16/ LO/1093). This study has been approved by the Health Research Authority and Health and Care Research Wales (IRAS Project ID 273001)

## Supplementary Information

Supplementary Information

## Data Availability

Requests can be made by researchers to access the NNRD through: https://www.imperial.ac.uk/neonatal-data-analysis-unit/neonatal-data-analysis-unit/utilising-the-national-neonatal-research-database/.
