# Supplementary material for: Identification of variation in nutritional practice in neonatal units in England and association with clinical outcomes using agnostic machine learning
Source: Sci Rep. 2021 Mar 30;11:7178. doi: 10.1038/s41598-021-85878-z (PMC8009880; doi:10.1038/s41598-021-85878-z)
Supplement: Supplementary file 1 — Supplementary Information [file 41598_2021_85878_MOESM1_ESM.docx]

## Supplementary information

**Table S1.** Statistics on the within-cluster populations for all the 46 nutritional patterns.

**Table S2.** Results on the reproducibility of DPGMM model fits over 10 pairs of runs on the whole cohort (n=45,679 infants).

**Table S3.** Statistical comparison between odd and even birth year sub-cohorts.

**Table S4.** Definition of the binary variable “NEC” corresponding to severe Necrotising Enterocolitis.

**Tables S5.** Contributing neonatal units and lead clinician.

**Fig. S1** Qualitative comparison on the selected 46 nutritional patterns: DPGMM posterior model versus the mean values observed within the clustered infants, using the whole cohort (n=45,679).

**Fig. S2** Exclusion criteria applied on the whole NNRD cohort.

**Table S1.** **Statistics on the within-cluster populations for all the 46 nutritional patterns**. Proportions, mean/median values are reported for admission, geographical location, outcome and nutritional variables as explained in Table 2. Proportions may not sum to 100% due to rounding, and 0% correspond to <0.5%. Colour coding is added to visually assist reading, with intensities scaled between min (brightest) and max (darkest) values over all clusters. Colour coding of admission/outcome variables: Green= “favourable” values, White= values close to whole-cohort average, Purple= “unfavourable” values. Colour coding of geographical location variables: Green= frequency < whole cohort, Purple= frequency > whole cohort. Colour coding of nutritional components: same as in Fig. 2.

| **Comparison** | **ELBO 1** | **ELBO 2** | **Number of clusters 1** | **Number of clusters 2** | **Number of clusters 1 (covering 80%)** | **Number of clusters 2 (covering 80%)** | **F-measure** | **F-measure (covering 80%)** |
| --- | --- | --- | --- | --- | --- | --- | --- | --- |
| 1 | 586,039 | 583,754 | 46 | 49 | 9 | 8 | 0.85 | 0.85 |
| 2 | 584,891 | 582,277 | 48 | 46 | 8 | 8 | 0.75 | 0.77 |
| 3 | 584,085 | 583,675 | 48 | 49 | 9 | 9 | 0.89 | 0.93 |
| 4 | 583,615 | 583,574 | 49 | 49 | 9 | 9 | 0.87 | 0.89 |
| 5 | 583,893 | 582,193 | 47 | 45 | 9 | 9 | 0.82 | 0.84 |
| 6 | 582,079 | 585,703 | 43 | 46 | 8 | 10 | 0.81 | 0.81 |
| 7 | 583,750 | 586,767 | 46 | 48 | 9 | 10 | 0.83 | 0.86 |
| 8 | 585,650 | 586,862 | 47 | 46 | 10 | 10 | 0.83 | 0.88 |
| 9 | 584,955 | 583,785 | 48 | 48 | 9 | 9 | 0.77 | 0.77 |
| 10 | 585,070 | 583,628 | 51 | 49 | 9 | 9 | 0.80 | 0.85 |
| **Mean** | **584,403** | **584,222** | **47** | **48** | **9** | **9** | **0.82** | **0.85** |

**Table S2.** **Results on the reproducibility of DPGMM model fits over 10 pairs of runs on the whole cohort (n=45,679 infants).** 20 independent runs using different stochastic seed initialisations (indices 1 and 2 in header names) are paired into 10 comparisons, numbered 1 to 10. For each pair of runs, the evidence lower bound (ELBO) metric, the total number of identified nutritional clusters, and the number of clusters covering 80% of the whole cohort are reported with indices 1 and 2. Reproducibility of whole cohort clustering results between pairs of runs is measured with the F-measure applied on all clusters and on clusters covering 80% of the whole cohort. Rounded mean values over the 10 comparisons are also reported for all variables.

|  | **Mean and Standard Deviation** | | | **p-values** | | |
| --- | --- | --- | --- | --- | --- | --- |
| **Variable** | **All** | **Odd** | **Even** | **All versus Odd** | **All versus Even** | **Odd versus Even** |
| **N** | 45,679 | 21,117 | 21,230 | **-** | **-** | **-** |
| **North** | 0.30 ± 0.46 | 0.30 ± 0.46 | 0.30 ± 0.46 | 0.676 | 0.543 | 0.378 |
| **Midlands** | 0.27 ± 0.44 | 0.26 ± 0.44 | 0.27 ± 0.45 | 0.132 | 0.265 | 0.025 |
| **London** | 0.21 ± 0.40 | 0.20 ± 0.40 | 0.21 ± 0.41 | 0.853 | 0.531 | 0.486 |
| **South** | 0.23 ± 0.42 | 0.23 ± 0.42 | 0.23 ± 0.42 | 0.192 | 0.263 | 0.038 |
| **Birth Year** | 2015 ± 2 | 2015 ± 2 | 2014 ± 2 | <0.001 | <0.001 | <0.001 |
| **Gestational Age (weeks)** | 29 ± 2 | 29 ± 2 | 29 ± 2 | 0.257 | 0.442 | 0.246 |
| **BW z-score** | -0.23 ± 0.94 | -0.24 ± 0.94 | -0.23 ± 0.94 | 0.186 | 0.174 | 0.058 |
| **Resuscitation** | 0.08 ± 0.28 | 0.08 ± 0.27 | 0.09 ± 0.28 | 0.621 | 0.425 | 0.265 |
| **Antenatal Steroids** | 0.90 ± 0.30 | 0.91 ± 0.29 | 0.90 ± 0.30 | 0.150 | 0.072 | 0.006 |
| **Sex (proportion of females)** | 0.46 ± 0.50 | 0.45 ± 0.50 | 0.46 ± 0.50 | 0.380 | 0.494 | 0.181 |
| **Apgar 1 min** | 6 ± 2 | 6 ± 2 | 6 ± 2 | 0.236 | 0.043 | 0.018 |
| **Apgar 5 min** | 8 ± 2 | 8 ± 2 | 8 ± 2 | 0.144 | 0.019 | 0.004 |
| **Apgar 10 min** | 9 ± 2 | 9 ± 2 | 9 ± 2 | 0.486 | 0.491 | 0.481 |
| **Mortality** | 0.08 ± 0.28 | 0.08 ± 0.27 | 0.09 ± 0.28 | 0.298 | 0.296 | 0.074 |
| **Necrotising Enterocolitis (NEC)** | 0.03 ± 0.18 | 0.03 ± 0.18 | 0.03 ± 0.18 | 0.851 | 0.797 | 0.957 |
| **Bronchopulmonary Dysplasia (BPD)** | 0.30 ± 0.46 | 0.30 ± 0.46 | 0.30 ± 0.46 | 0.985 | 0.555 | 0.624 |
| **Maternal Milk at Discharge** | 0.56 ± 0.50 | 0.56 ± 0.50 | 0.56 ± 0.50 | 1.000 | 0.759 | 0.795 |
| **Length of Stay (days)** | 57 ± 37 | 57 ± 36 | 57 ± 38 | 0.432 | 0.328 | 0.407 |
| **W36 z-score** | -1.59 ± 0.95 | -1.59 ± 0.95 | -1.60 ± 0.96 | 0.431 | 0.116 | 0.121 |
| **W36dz** | -1.20 ± 0.75 | -1.19 ± 0.74 | -1.22 ± 0.77 | 0.151 | 0.009 | 0.002 |
| **Maternal Milk** | 0.66 ± 0.35 | 0.67 ± 0.34 | 0.66 ± 0.35 | 0.029 | 0.001 | <0.001 |
| **Human Donor Milk** | 0.06 ± 0.13 | 0.06 ± 0.13 | 0.05 ± 0.13 | 0.091 | <0.001 | <0.001 |
| **Breast Milk Fortifier** | 0.14 ± 0.20 | 0.14 ± 0.20 | 0.13 ± 0.20 | 0.339 | 0.008 | 0.008 |
| **Formula Milk** | 0.39 ± 0.34 | 0.39 ± 0.34 | 0.38 ± 0.34 | 0.428 | 0.407 | 0.361 |
| **Parenteral Nutrition** | 0.21 ± 0.22 | 0.21 ± 0.22 | 0.21 ± 0.22 | 0.032 | 0.002 | <0.001 |
| **Glucose Electrolyte** | 0.17 ± 0.21 | 0.17 ± 0.20 | 0.17 ± 0.21 | 0.171 | 0.057 | 0.015 |

**Table S3.** **Statistical** **comparison between odd and even birth year sub-cohorts.** P-values are reported for Mann-Whitney U tests on variables derived from continuous entries (e.g. Birth year, z-scores) and Fisher’s Exact Test on variables derived from binary values (e.g. Geographical location, Resuscitation, BPD). Mean values are reported for each variable.

**Table S4.** **Definition of the binary variable “NEC” corresponding to severe Necrotising Enterocolitis.** Names of NNRD tables and variables are listed, along with the required values to characterize severe NEC.

**Table S5.** **Contributing neonatal unit and lead clinician.**

| **Institution** | **Lead** |
| --- | --- |
| Airedale General Hospital | Dr Matthew Babirecki |
| Arrowe Park Hospital | Dr Anand Kamalanathan |
| Barnet Hospital | Dr Tim Wickham |
| Barnsley District General Hospital | Dr Kavi Aucharaz |
| Basildon Hospital | Dr Aashish Gupta |
| Basingstoke & North Hampshire Hospital | Dr Nicola Paul |
| Bassetlaw District General Hospital | Dr L M Wong |
| Bedford Hospital | Dr Anita Mittal |
| Birmingham City Hospital | Dr Lindsay Halpern |
| Birmingham Heartlands Hospital | Dr Pinki Surana |
| Birmingham Women's Hospital | Dr Matt Nash |
| Bradford Royal Infirmary | Dr Sunita Seal |
| Broomfield Hospital, Chelmsford | Dr Ahmed Hassan |
| Calderdale Royal Hospital | Dr Karin Schwarz |
| Chelsea & Westminster Hospital | Dr Shu-Ling Chuang |
| Chesterfield & North Derbyshire Royal Hospital | Dr Aiwyne Foo |
| Colchester General Hospital | Dr Jo Anderson |
| Conquest Hospital | Dr Graham Whincup |
| Countess of Chester Hospital | Dr Stephen Brearey |
| Croydon University Hospital | Dr John Chang |
| Cumberland Infirmary | Dr Yee Aung |
| Darent Valley Hospital | Dr Abdul Hasib |
| Darlington Memorial Hospital | Dr Mehdi Garbash |
| Derriford Hospital | Dr Alex Allwood |
| Diana Princess of Wales Hospital | Dr Pauline Adiotomre |
| Doncaster Royal Infirmary | Dr Nigel Brooke |
| Dorset County Hospital | Dr Abby Deketelaere |
| East Surrey Hospital | Dr K Abdul Khader |
| Epsom General Hospital | Dr Ruth Shephard |
| Frimley Park Hospital | Dr Sanghavi Rekha |
| Furness General Hospital | Dr Anas Olabi |
| George Eliot Hospital | Dr Mukta Jain |
| Gloucester Royal Hospital | Dr Jennifer Holman |
| Good Hope Hospital | Dr Pinki Surana |
| Great Western Hospital | Dr Stanley Zengeya |
| Guy's & St Thomas' Hospital | Dr Geraint Lee |
| Harrogate District Hospital | Dr Sobia Balal |
| Hereford County Hospital | Dr Cath Seagrave |
| Hillingdon Hospital | Dr Tristan Bate |
| Hinchingbrooke Hospital | Dr Hilary Dixon |
| Homerton Hospital | Dr Narendra Aladangady |
| Hull Royal infirmary | Dr Hassan Gaili |
| Ipswich Hospital | Dr Matthew James |
| James Cook University Hospital | Dr M Lal |
| James Paget Hospital | Dr Ambadkar |
| Kettering General Hospital | Dr Poornima Pandey |
| Kings College Hospital | Dr Ravindra Bhat |
| King's Mill Hospital | Dr Simon Rhodes |
| Kingston Hospital | Dr Vinay Pai |
| Lancashire Women and Newborn Centre | Dr Savi Sivashankar |
| Leeds Neonatal Service | Dr Lawrence Miall |
| Leicester General Hospital | Dr Jonathan Cusack |
| Leicester Royal Infirmary | Dr Venkatesh Kairamkonda |
| Leighton Hospital | Dr Michael Grosdenier |
| Lincoln County Hospital | Dr Ajay Reddy |
| Lister Hospital | Dr J Kefas |
| Liverpool Women's Hospital | Dr Christopher Dewhurst |
| Luton & Dunstable Hospital | Dr Jennifer Birch |
| Macclesfield District General Hospital | Dr Gail Whitehead |
| Manor Hospital | Dr Krishnamurthy |
| Medway Maritime Hospital | Dr Ghada Ramadan |
| Milton Keynes General Hospital | Dr I Misra |
| Musgrove Park Hospital | Dr Chris Knight |
| New Cross Hospital | Dr Rob Negrine |
| Newham General Hospital | Dr Imdad Ali |
| Nobles Hospital | Dr Prakash Thiagarajan |
| Norfolk & Norwich University Hospital | Dr Mark Dyke |
| North Devon District Hospital | Dr Michael Selter |
| North Manchester General Hospital | Dr P Kamath |
| North Middlesex University Hospital | Dr Neeraj Jain |
| Northampton General Hospital | Dr Subodh Gupta |
| Northumbria Specialist Emergency Care Hospital | Laura Winder |
| Northwick Park Hospital | Dr Richard Nicholl |
| Nottingham City Hospital | Dr Steven Wardle |
| Nottingham University Hospital (QMC) | Dr Steven Wardle |
| Ormskirk District General Hospital | Dr Andreea Bontea |
| Oxford University Hospitals, John Radcliffe Hospital | Dr Eleri Adams |
| Peterborough City Hospital | Dr Katharine McDevitt |
| Pilgrim Hospital | Dr Ajay Reddy |
| Pinderfields General Hospital | Dr David Gibson |
| Poole General Hospital | Prof Minesh Khashu |
| Princess Alexandra Hospital | Dr Chinnappa Reddy |
| Princess Anne Hospital | Dr Mark Johnson |
| Princess Royal Hospital | Dr P Amess |
| Princess Royal Hospital | Dr Deshpande |
| Princess Royal University Hospital | Dr Elizabeth Sleight |
| Queen Alexandra Hospital | Dr Charlotte Groves |
| Queen Charlotte's Hospital | Dr Lidia Tyszcuzk |
| Queen Elizabeth Hospital, Gateshead | Dr Anne Dale |
| Queen Elizabeth Hospital, King's Lynn | Dr Glynis Rewitzky |
| Queen Elizabeth Hospital, Woolwich | Dr Olutoyin Banjoko |
| Queen Elizabeth the Queen Mother Hospital | Dr Bushra Abdul-Malik |
| Queen's Hospital, Burton on Trent | Dr Dominic Muogbo |
| Queen's Hospital, Romford | Dr Khalid Mannan |
| Rosie Maternity Hospital, Addenbrookes | Dr Angela D'Amore |
| Rotherham District General Hospital | Dr Shameel Mattara |
| Royal Albert Edward Infirmary | Dr Christos Zipitis |
| Royal Berkshire Hospital | Dr Peter De Halpert |
| Royal Bolton Hospital | Dr Paul Settle |
| Royal Cornwall Hospital | Dr Paul Munyard |
| Royal Derby Hospital | Dr John McIntyre |
| Royal Devon & Exeter Hospital | Dr Chrissie Oliver |
| Royal Hampshire County Hospital | Dr Lucinda Winckworth |
| Royal Lancaster Infirmary | Dr Joanne Fedee |
| Royal Oldham Hospital | Dr Natasha Maddock |
| Royal Preston Hospital | Dr Richa Gupta |
| Royal Stoke University Hospital | Dr Jyoti Kapur |
| Royal Surrey County Hospital | Dr Ben Obi |
| Royal Sussex County Hospital | Dr P Amess |
| Royal United Hospital | Dr Stephen Jones |
| Royal Victoria Infirmary | Dr Naveen Athiraman |
| Russells Hall Hospital | Dr Chandan Gupta |
| Salisbury District Hospital | Dr Jim Baird |
| Scarborough General Hospital | Dr Kirsten Mack |
| Scunthorpe General Hospital | Dr Pauline Adiotomre |
| Southend Hospital | Dr Vineet Gupta |
| Southmead Hospital | Dr Alison Pike |
| St George's Hospital | Dr Charlotte Huddy |
| St Helier Hospital | Dr Ralf Hartung |
| St Mary's Hospital, Isle of Wight | Dr Akinsola Ogundiya |
| St Mary's Hospital, London | Dr Lidia Tyszcuzk |
| St Mary's Hospital, Manchester | Dr Ngozi Edi-Osagie |
| St Michael's Hospital | Dr Pamela Cairns |
| St Peter's Hospital | Dr Peter Martin |
| St Richard's Hospital | Dr Nick Brennan |
| Stepping Hill Hospital | Dr Carrie Heal |
| Stoke Mandeville Hospital | Dr Sanjay Salgia |
| Sunderland Royal Hospital | Dr Majd Abu-Harb |
| Tameside General Hospital | Dr Jacqeline Birch |
| The Jessop Wing, Sheffield | Dr Porus Bastani |
| The Royal Free Hospital | Dr Marice Theron |
| The Royal London Hospital | Dr Vadivelam Murthy |
| Torbay Hospital | Dr Siba Paul |
| Tunbridge Wells Hospital | Dr Hamudi Kisat |
| University College Hospital | Dr Giles Kendall |
| University Hospital Coventry | Dr Puneet Nath |
| University Hospital Lewisham | Dr Ozioma Obi |
| University Hospital of North Durham | Dr Mehdi Garbash |
| University Hospital of North Tees | Dr Hari Kumar |
| Victoria Hospital, Blackpool | Dr Chris Rawlingson |
| Warrington Hospital | Dr Delyth Webb |
| Warwick Hospital | Dr Bird |
| Watford General Hospital | Dr Sankara Narayanan |
| West Cumberland Hospital | Dr Yee Mon Aung |
| West Middlesex University Hospital | Dr Eleanor Hulse |
| West Suffolk Hospital | Dr Ian Evans |
| Wexham Park Hospital | Dr Rekha Sanghavi |
| Whipps Cross University Hospital | Dr Caroline Sullivan |
| Whiston Hospital | Dr Ros Garr |
| Whittington Hospital | Dr Wynne Leith |
| William Harvey Hospital | Dr Vimal Vasu |
| Worcestershire Royal Hospital | Dr Liza Harry |
| Worthing Hospital | Dr Katia Vamvakiti |
| Wythenshawe Hospital | Dr Ngozi Edi-Osagie |
| Yeovil District Hospital | Dr Megan Eaton |
| York District Hospital | Dr Sundeep Sandhu |

**Fig. S1** **Qualitative comparison on the selected 46 nutritional patterns: DPGMM posterior model versus the mean values observed within the clustered infants, using the whole cohort (n=45,679).** Nutritional patterns are encoded as the proportion of days over the total time in care a nutritional component was given (between 0 and 1). Clusters are ranked by size. Colour intensities encode values. MM = Maternal Milk, HDM = Human Donor Milk, BMF = Breast Milk Fortifier FM= Formula milk, PN Parenteral Nutrition, GE=Glucose Electrolyte solution.

**Fig. S2** **Exclusion criteria applied on the whole NNRD cohort.** The final cohort comprised 45,679 infants and 2,535,062 daily records of care.
